# Receptor-binding domain-anchored peptides block binding of severe acute respiratory syndrome coronavirus 2 spike proteins with cell surface angiotensin-converting enzyme 2

**DOI:** 10.3389/fmicb.2022.910343

**Published:** 2022-09-13

**Authors:** Ting Wang, Jie Xu, Beibei Wang, Yulian Wang, Wei Zhao, Bin Xiang, Yuhua Xue, Quan Yuan, Yiqiang Wang

**Affiliations:** ^1^Oncology Department of Integrated Traditional Chinese and Western Medicine, The First Affiliated Hospital of Anhui Medical University, Hefei, Anhui, China; ^2^Central Laboratory, Xiang’an Hospital of Xiamen University, Xiang’an University Medical Center, Xiamen University, Xiamen, Fujian, China; ^3^Eye Institute of Xiamen University, Medical College, Xiamen University, Xiamen, Fujian, China; ^4^Center for Advanced Materials Research, Advanced Institute of Natural Sciences, Beijing Normal University, Zhuhai, Guangdong, China; ^5^Ministry of Education (MOE) Key Laboratory of Protein Sciences, Department of Basic Medical Sciences, School of Medicine, Tsinghua University, Beijing, China; ^6^Fujian Provincial Key Laboratory of Innovative Drug Target Research, School of Pharmaceutical Sciences, Xiamen University, Xiamen, Fujian, China; ^7^State Key Laboratory of Molecular Vaccinology and Molecular Diagnostics, School of Public Health, Xiamen University, Xiamen, Fujian, China; ^8^Wisdom Lake Academy of Pharmacy, Xi’an Jiaotong-Liverpool University, Suzhou, Jiangsu, China

**Keywords:** SARS-CoV-2, spike protein, ACE2, RBD domain, phage display

## Abstract

**Background:**

The COVID-19 pandemic has killed over 6 million people worldwide. Despite the accumulation of knowledge about the causative pathogen severe acute respiratory syndrome coronavirus 2 (SARS-CoV-2) and the pathogenesis of this disease, cures remain to be discovered. We searched for certain peptides that might interfere with spike protein (S protein)-angiotensin-converting enzyme 2 (ACE2) interactions.

**Methods:**

Phage display (PhD)-12 peptide library was screened against recombinant spike trimer (S-trimer) or receptor-binding domain (S-RBD) proteins. The resulting enriched peptide sequences were obtained, and their potential binding sites on S-trimer and S-RBD 3D structure models were searched. Synthetic peptides corresponding to these and other reference sequences were tested for their efficacy in blocking the binding of S-trimer protein onto recombinant ACE2 proteins or ACE2-overexpressing cells.

**Results:**

After three rounds of phage selections, two peptide sequences (C2, DHAQRYGAGHSG; C6, HWKAVNWLKPWT) were enriched by S-RBD, but only C2 was present in S-trimer selected phages. When the 3D structures of static monomeric S-RBD (6M17) and S-trimer (6ZGE, 6ZGG, 7CAI, and 7CAK, each with different status of S-RBDs in the three monomer S proteins) were scanned for potential binding sites of C2 and C6 peptides, C6 opt to bind the saddle of S-RBD in both 6M17 and erected S-RBD in S-trimers, but C2 failed to cluster there in the S-trimers. In the competitive S-trimer-ACE2-binding experiments, synthetic C2 and C6 peptides inhibited S-trimer binding onto 293T-ACE2hR cells at high concentrations (50 μM) but not at lower concentrations (10 μM and below), neither for the settings of S-trimer binding onto recombinant ACE2 proteins.

**Conclusion:**

Using PhD methodology, two peptides were generated bearing potentials to interfere with S protein-ACE2 interaction, which might be further exploited to produce peptidomimetics that block the attachment of SARS-CoV-2 virus onto host cells, hence diminishing the pathogenesis of COVID-19.

## Introduction

The COVID-19 pandemic had caused 6.35 million death worldwide as of 8 July 2022 and still poses a serious challenge in some nations. Worse was that over two more folds of “excess deaths” might have occurred due to indirect consequences of the pandemic, such as changes in “social, economic, and behavioral responses to the pandemic, including strict lockdowns” (Collaborators, 2022). While vaccinations and natural infections build herd immunity that helps to protect people from infection or prevent pandemic recurrence (Lipsitch and Dean, 2020; Mobarak et al., 2022), cures are still lacking for the infected individuals in most areas. Among the scientific efforts, various therapeutics have been tried, such as cells (Sang et al., 2021), engineered antibodies (Matthay and Luetkemeyer, 2021), natural products (Bhattacharya and Paul, 2021), synthetical biologicals (Robson, 2020), and small molecules (Tiwari et al., 2020). Intended targets included viral structural proteins (Sheward et al., 2022), host products [e.g., interleukin 6 (IL-6)] (Murthy and Lee, 2021), viral replication process (Schafer et al., 2022), or host-virus interactions (Gordon et al., 2020). The strategies aiming at the first step of virus-host interactions sound most attractive. The viral spike (S) protein trimers (S-trimers) are thought to be the main molecules mediating the affinity of exogenous severe acute respiratory syndrome coronavirus 2 (SARS-CoV-2) virus for angiotensin-converting enzyme 2 (ACE2) or other less-attended molecules, such as TMPRSS2 on host cells (Hoffmann et al., 2020). Since the structures of both S and ACE2 proteins are known, computation or computer-based methods are thought to be high for novel drug discovery (Cao et al., 2020; Jawad et al., 2021). However, though a few candidates had been proposed in these *in silico* studies, only part of them had been proven effective in functional experimental studies, highlighting the demand for more robust strategies that mimic the actual virus-host interactions more faithfully.

Phage display (PhD) methodology, as exemplified in other infectious diseases (Huang et al., 2012; Alfaleh et al., 2020; Roth et al., 2021), met this end and has been tried in the context of SARS-CoV-2 or COVID-19. In detail, PhD has been successful in producing antibodies for neutralizing or detection (Noy-Porat et al., 2020; Bertoglio et al., 2021) in identifying COVID-19-induced antibodies to the virus (Zhao et al., 2021) or in searching for viral epitopes responsible for virus escaping immune responses (Garrett et al., 2021). Based on our previous experience using PhD in studies of the host-pathogen interactions (Zhao et al., 2012; Shen et al., 2017), we performed PhD screening to search for peptides that would bind the receptor-binding domain (RBD) domain of SARS-CoV-2 S protein (S-RBD). Theoretically, if such peptides could bind the site(s) critical for S-RBD interaction with its receptors (e.g., ACE2 or other molecules), they should interfere with S-trimer-ACE2 interactions. Furthermore, such an S-protein Entrapped Affinity Ligand (SEAL) peptide should be able to block the binding of the viruses with their target cells. Here, we report that two SEAL peptides were obtained *via* phage displaying against S-RBD and S-trimer proteins, and preliminary functional studies demonstrated weak blocking effects at high concentrations. Encouragingly, while this project was ongoing, three groups reported their results obtained by protocols mainly relying on PhD (Petrenko et al., 2022; Sevenich et al., 2022; Yang et al., 2022). The promises and limitations of these studies were also discussed.

## Materials and methods

### Phage display screening against spike receptor-binding domain or spike trimer proteins and confirmation of affinity of promising phages

Recombinant SARS-CoV-2 S-trimer proteins were from the commercial resource (Cat# DRA49, MW 136.6 kDa; Novoprotein Company, Suzhou, China), and recombinant S-RBD products corresponding to aa319-541 of YP_009724390.1, MW 30.7 kDa (Lan et al., 2020) was a generous gift from Li (Tsinghua University, Beijing, China). PhD-12 Peptide PhD Library Kit (New England BioLabs, Beverly, MA, United States) was used for PhD screening against these two proteins. Briefly, S-trimer proteins were immobilized overnight at 4°C on the enzyme-linked immunosorbent assay (ELISA) plates at 100 μg/ml in 0.1 M NaHCO_3_, pH 8.6. The plates were then blocked with 0.5% bovine serum albumin (BSA) in 0.1 M NaHCO_3_ buffer (containing 0.02% NaN_3_) for 1 h. After six washes with tris base-buffered saline solution (TBST buffer containing 0.01% Tween-20), 2 × 10^11^ phages in TBST buffer were added for 45 min at room temperature. After ten washes, bound phages were recovered, amplified in *Escherichia coli*, harvested into TBS buffer (containing 0.02% NaN_3_), quantified with a plaque-forming assay, and used the product for the second round display. After two or three screening rounds, bound phages were harvested into elution buffer (0.2 M Glycine-HCl, 1 mg/ml BSA, pH 2.2) and neutralized with 1 M tris base-HCl buffer, pH 9.1. After dilution, the phage mix was applied onto bacterial plates to obtain blue plaques. Thirty (after the second round) or 25 (after the third round) isolated plaques were randomly picked for phage DNA sequencing using the primers in the kit. The resulting 12-amino acids peptides translated from phage DNA inserts were analyzed, and the most promising sequence was used for subsequent studies.

Enzyme-linked immunoassay (ELISA) was used to confirm the affinity of the resulting monoclonal phages for targeted proteins. ELISA plates (Corning, NY, United States) were coated with 10 μg/ml S-trimer or S-RBD proteins. With the starting original library phages (O virions) as control, all selected interest phages were amplified, titrated, and added to the plates at different concentrations (2.5 × 10^9^, 1 × 10^10^, 4 × 10^10^, 1.6 × 10^11^, and 6.4 × 10^11^ phage virions in 100 μl) for 1 h at room temperature. The plates were washed six times with TBST washing buffer and then incubated with diluted horseradish peroxidase (HRP)-conjugated anti-M13 monoclonal antibody (GE Healthcare, Piscataway, NJ, United States) for 1 h. After six washes, 3,3′, 5,5-tetramethylbenzidine (TMB) solution (Beyotime Biotechnology, Shanghai, China) was added to the plate, and after 10 min of development, the reaction was stopped by adding 2 M H_2_SO_4_ solution. Optical absorbance was measured at 450 nm in a microplate reader (Synergy H1, BioTek Instruments, Inc., Winooski, VT, United States).

### PEP-SiteFinder modeling of candidate spike-protein entrapped affinity ligand peptides docking onto monomeric receptor-binding domain or spike trimer proteins

The surface of RBD or S-trimer proteins was scanned using the PEP-SiteFinder (Saladin et al., 2014). The 3D models of RBD to locate the potential docking site(s) of interest peptide(s) on S proteins (Yan et al., 2020) or S-trimers (Lv et al., 2020; Wrobel et al., 2020) were retrieved from Research Collaboratory for Structural Bioinformatics Protein Data Bank (RCSB PDB). The top 50 poses of each peptide in each protein model were checked to identify the most likely binding site(s). Cn3D (Wang et al., 2000) was also utilized for viewing these protein structures.

### Measurement of the effect of synthetic peptides on spike trimer-angiotensin-converting enzyme 2 binding

293T-ACE2hR cells, a cell line consistently expressing human ACE2 (hACE2) on the cell surface (Supplementary Figure 1; Zhang et al., 2021), or recombinant ACE2 proteins (Cat#10108-H02H, Novoprotein) were utilized to test the potential effect of interest peptides on S-trimer-ACE2 binding. In brief, two possible SEAL peptides derived from the above analysis (C2 and C6) and two reference peptides [spike-binding peptide 1 (SBP1) and spike-binding peptide 1 (SBP2)] (Zhang et al., 2020) were ordered from Biotech Bioscience and Technology (Shanghai, China) and dissolved in PBS. Their sequences were as follows: C2, DHAQRYGAGHSG; C6, HWKAVNWLKPWT; SBP1, IEEQAKTFLDKFNHEAEDLFYQSK; and SBP2, TFLDKFNHEAED. 293T-ACE2hR cells were grown in 96-well plates until confluent in the first measurement setting. S-trimer at 2 nM was mixed with equal volume (25 μl) of peptides at different concentrations (0, 0.16, 0.8, 4, 20, and 100 μM) and kept at room temperature for 1 h. After removing the culture medium from the cells, the mixture was added (50 μl/well) and kept at room temperature for 1 h. Unbound peptides and proteins were removed, and the cells were washed three times with PBS. HRP-conjugated Anti-6X His tag^®^ antibody (diluted at 1:10,000; Abcam, Cambridge, MA, United States) was added to each well for 1 h at room temperature. After three washes with PBS, TMB Solution (Beyotime Biotechnology) was added to the plate, and the plate was read at OD370 nm in a Multiskan Go Spectrophotometer (Thermo Fisher Scientific, Waltham, MA, United States).

Then, 293T-ACE2hR cells were substituted by recombinant ACE2 proteins and coated onto ELISA plates in the other measurement setting. In brief, recombinant ACE2 proteins (Novoprotein) were immobilized overnight at room temperature on ELISA plates at a 5 μg/ml concentration in 0.1 M NaHCO_3_, pH 9.6. The plates were then blocked with 10% fetal calf serum for 2 h and washed with tris-buffered saline solution containing 0.1% Tween-20 [phosphate-buffered saline (PBST) buffer]. Then a premixture of S-trimer proteins (final concentration 0.01 μM) with peptides (C2, C6, SBP1, and SBP2) of different concentrations (0.5, 1.65, and 5 μM) was added to each well (50 μl/well). The following procedures were described above for the 293T-ACE2hR cells setting.

## Results and discussion

### Obtainment of two promising spike-protein entrapped affinity ligand peptide sequences displayed against spike-receptor-binding domain

**FIGURE 1:**
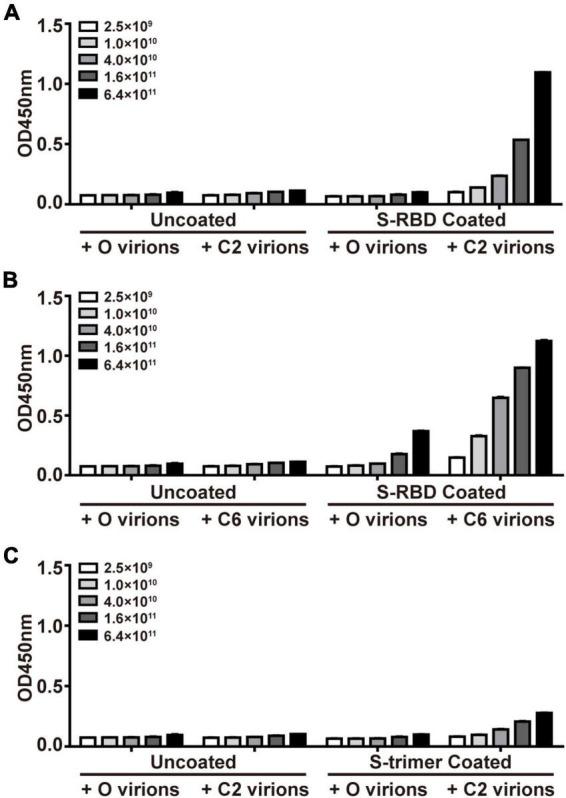
Confirmation of affinity binding of phages of S-protein Entrapped Affinity Ligand (SEAL) sequences with recombinant spike-receptor-binding domain (S-RBD) **(A,B)** or S-trimer proteins **(C)**. The plates were coated with S-RBD (10 μg/ml, i.e., about 0.33 μM, **A,B**) or S-trimer proteins (10 μg/ml, i.e., about 0.073 μM, **C**). The interest phages (C2 and C6) and starting library phages (O) were serially diluted and tested for binding. The apparent less efficient C2 virions to bind S-trimer **(C)** than to bind S-RBD **(A)** might be due to these two targets’ difference in molar concentrations (by about 4.4-fold) at the same mass concentrations.

Phage display has been widely used in identifying interacting partners of target molecules that were included in previous projects of this team (Zhao et al., 2012; Wang et al., 2019). In the current study, we applied PhD on S-trimers or S-RBD proteins, aiming to obtain peptides supposedly able to “seal” the potent binding site on their surface. After three rounds of panning PhD against recombinant S-RBD proteins, two phages with peptide sequences DHAQRYGAGHSG (C2) and HWKAVNWLKPWT (C6) were enriched in the elutes, each of them accounting for 10 clones in all 25 sequenced clones. Interestingly, C2 and C6 accounted for 7 and 6 clones in the elute after the second panning in all 30 sequenced clones. Therefore, we did not attempt more rounds of panning. When S-trimer proteins were used for panning, only the C2 sequence dominated the elutes, accounting for 20 of 30 clones after the second panning and 16 of 25 phages after the third panning, respectively. Next, using the starting library phages (O virions) as control, the ELISA assay demonstrated dose-dependent binding of monoclonal C2 and C6 phages to immobilized S-RBD proteins and C2 phages for S-trimer protein (Figure 1). An accurate comparison between affinity of C2 and C6 phages for the same target (e.g., S-RBD proteins) was not attempted, or between affinities of C2 phages for different target proteins (i.e., S-RBD or S-trimer). Measurement of affinity of C6 phages for S-trimer was not attempted either.

### Three-dimensional modeling of peptides binding onto spike receptor-binding domain or spike trimer

**FIGURE 2:**
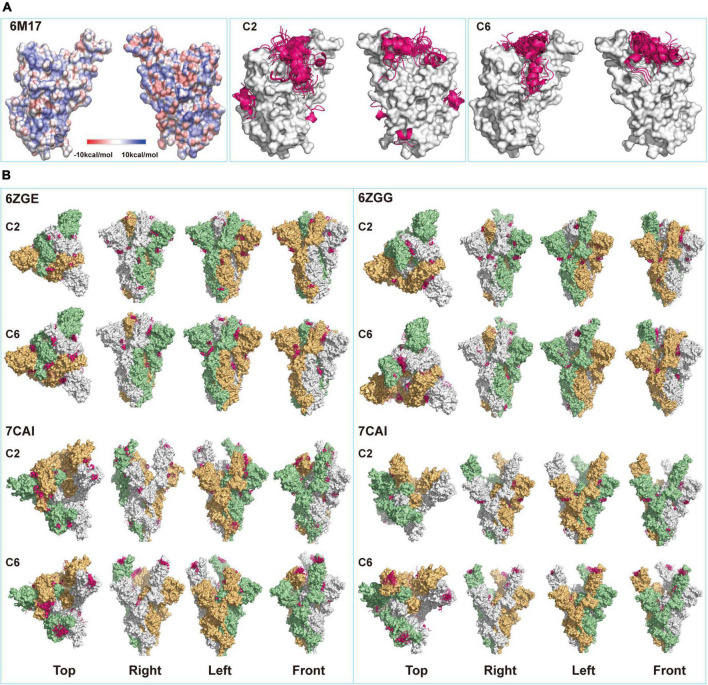
Structural modeling of peptides-spike interactions. **(A)** Comparing binding patterns for C2 and C6 peptides (pink belts) onto spike-receptor-binding domain (S-RBD). Shown are the fifty poses with the highest of each binding exclusively onto the saddle of RBD (6M17, open), some poses of C2 peptide bind onto other sites on the surface of RBD. **(B)** Four configurations corresponding to the different status of RBDs in the S-trimers (6ZGE, 6ZGG, 7CAI, and 7CAK for 0, 1, 2, and 3 RBDs open, respectively) were compared for their potential bindings with the two peptides. Each figuration was given a view from the top, right, left, and front sides.

Previous crystal structural studies suggested that the resting S-trimers on the virus surface took a “closed” figuration and, upon contacting ACE2 (or other receptors) on host cells, went through the opening process and exposed the RBD (Wrobel et al., 2020). Surely, this opening process would also alter the configuration of the whole molecule. When the static model of single S-RBD protein (6M17) was used for predicting peptide binding sites, it was found that all fifty C6 peptide poses were docked onto the saddle of S-RBD, while a fraction of C2 peptide poses were docked onto other sites that were supposedly not to directly affect S-RBD’s receptor binding functions (Figure 2A). When S-trimer was used for modeling, binding sites for C2 poses were even more dispersed, and few of them would dock onto S-RBD saddlebacks, independent of S-trimers’ configuration (Figure 2B). For example, in the all-closing status of the S-trimer (6ZGE), 11 of 50 C2 poses were located in the spaces among the three S-RBDs, 6 between neighboring S-RBDs, and 8 in the middle between S-RBD and N-terminal domain (NTD). In the all-open configuration (7CAK), only 6 poses were related to S-RBD and not any poses were docked onto the saddle of open S-RBDs.

On the contrary, once a single S-RBD was erected (6ZGG), several C6 poses were docked onto its saddle. When one or two more S-RBDs were erected (7CAI and 7CAK, respectively), almost all C6 poses were on their saddlebacks. We assumed that in an actual environment that contained both virus and host cells, if the C2 or C6 peptides were present when S-trimers were in the closed configuration just like in resting virus, their binding onto the surface of S-trimers might facilitate or hinder the opening or erection of S-RBD, which deserved in-depth investigation in future. However, once the S-trimer opening process was initiated, C6 peptides should be able to bind the saddle of S-RBDs. Since it has been well documented that the saddle section was critical for RBD functions, such as binding with ACE2 (Lan et al., 2020; Wang et al., 2020) and being immunogenic (Greaney et al., 2021), we proposed that C6 peptides might interfere with the interaction of S-trimer with ACE2 by SEALing the S-RBD saddle(s). The differential docking sites’ prediction observed for C2 and C6 might partially explain that C2 virions were enriched by both S-RBD and S-trimer proteins, while the C6 virions were enriched by S-RBD proteins only (Figure 1).

### Synthetic C2 and C6 peptides blocked the binding of spike-trimers onto 293T-ACE2hR cells

**FIGURE 3:**
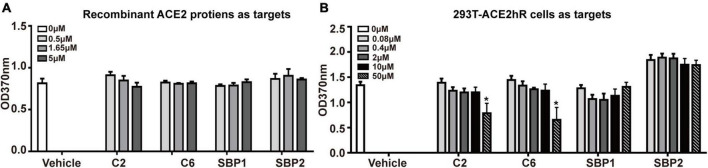
The potential blocking effect of peptides on the binding of S-trimer onto cellular or soluble angiotensin-converting enzyme 2 (ACE2). **(A)** The enzyme-linked immunoassay (ELISA) was applied to assess the blocking ability of synthetic peptides C2 and C6 at binding between ACE2 molecules and S-trimer protein, and the peptide SBP1 was used as positive control and peptide spike-binding peptide 2 (SBP2) as the negative control. **(B)** ELISA was applied to assess the blocking ability of synthetic peptides C2 and C6 at binding between 293T-ACE2hR cells and S-trimer protein. The peptide SBP1 was used as a positive and peptide SBP2 as the negative control. *p < 0.05, vs. vehicle, all by Student’s *t*-test.

When the above hypothesis was tested on recombinant ACE2 proteins coated on a solid surface, no blocking effect was observed for any tested peptides even at a 500:1 (5 μM vs. 0.01 μM, peptide vs. S-trimer) ratio (Figure 3A). These peptides’ ineffectiveness in blocking S-trimer binding with ACE2 at equivalent concentrations was also observed when 293T-ACE2hR cells were used as the source of S-trimer targets in living cells. When 50,000-fold overdose of C2 or C6 peptides was present, namely, 50 μM peptides vs. 0.001 μM S-trimer proteins, a blocking effect was observed for C2 and C6, but still not for SBP1 or SBP2 peptides (Figure 3B).

Failure of 500-fold overdose of C2 or C6 peptides to block S-trimers binding with their receptors was disappointing, and we suggested two possible explanations for this failure, especially for C6. First, the occupation of the saddle by C6 did not cause enough stereotype blockade as expected, which might be due to the small size of this 12-aa peptide, especially when most poses lay on the saddle “seat” in a vertical-crossing orientation rather than paralleling along the saddle axis (Figure 2A). It has been demonstrated that the “cantle” and “pommel” contributed more than the “seat” (Supplementary Figure 2) to the overall affinity and configuration fitting between S-RBD and ACE2 (Lan et al., 2020; Wang et al., 2020). Second, the affinity of these peptides for S-RBD might be too low to constitute effective competitors when natural and intact receptors (i.e., ACE2 molecules) were present, which might be the case for the SBP1 and SBP2 peptides. SBP1 was from the N-terminal sequence of hACE2 (e.g., α-helix 1) that was supposed to contact S-RBD if it intact ACE2 (Supplementary Figure 2; Zhang et al., 2020). Though those investigators demonstrated the association of SBP1 peptides with S-RBD proteins at the level of micromolar scales using bio-layer interferometry, SBP1 peptides neither associated with cell surface S-RBD proteins nor did they outcompete ACE2 binding onto S-RBD proteins (Zhang et al., 2020). More rigorous studies should verify such explanations and determine the associated factors between C6 and C2 peptides for RBD or S-trimers.

Another issue deserving discussion was why C2 and C6 behaved similarly in the competition assay (Figure 3) though they manifested different enrichment patterns in PhD panning (Figure 1), as well as different binding properties in the 3D modeling assay (Figure 2). It was known that configuration changes of S-trimers when virus encountered host components were critical for higher affinity interactions between S-trimers and ACE2 molecules. It was understandable that such interactions would be unique in many aspects and depend on the dynamic minutiae of all parties, and we would arbitrarily assume that the factors causing differential binding patterns of C2 or C6 virions onto S-RBD/S-trimers in PhD assays did not contribute enough interference to the actual interactions between S-trimers and ACE2 in the competition context, which are reflected in Figure 3.

Lastly, as a useful tool for studying molecular interactions at both biophysical and functional levels, PhD has been successful in identifying peptides that might be directly utilized to block pathogen invasions in this lab (Zhao et al., 2012) or others (Hall et al., 2009; Wei et al., 2020). This study and several other efforts (Anand et al., 2021; Sokullu et al., 2021; Ballmann et al., 2022; Labriola et al., 2022; Petrenko et al., 2022; Sevenich et al., 2022; Yang et al., 2022) demonstrated that PhD was a plausible method for generating possible therapeutics to treat COVID-19 as well. For example, Petrenko used phage-displayed spike S1 protein mimotopes to search for “all” cellular receptors, including authentic and alternative ones. Interestingly, FGFR3 was identified as an alternative receptor to S proteins (Petrenko et al., 2022). Since FGFR3 manifested a distribution pattern different from that of ACE2, Petrenko’s work expanded the area of SARS-CoV-2 targets and might lead to the discovery of novel pathogenesis of SARS-CoV-2. Like us, Sevenich performed three rounds of screening on S-RBD proteins using a 16-aa phage library combined with high throughput sequencing. The five final sequences they obtained manifested variable affinity for S-RBD proteins in confirmation assays, with Kd from 1.3 to 89.4 μM (Sevenich et al., 2022). Others also demonstrated the stringent dependence of efficacy of intended therapeutics on their molecular compositions and configuration (Raghuvamsi et al., 2021). With the help of computation of RBD-ACE2 interactions, the Baker’s team generated mini-proteins of 56–64 residues with inhibitory concentrations of 24–35 nM for RBD-ACE2 binding (Cao et al., 2020). On the contrary, also based on analysis of motifs or amino acids involved in RBD-ACE2 interaction, Chitsike proposed six peptides (20–29 aa) mimicking S-RBD fragment or hACE2 fragment but found that their IC50 for inhibiting RBD-ACE2 binding in experiments varied from 27 to 363 μM (Chitsike et al., 2021). For us, the direction of our future study would be to use the current C6 SEAL peptides as the core sequences and to develop them into larger molecules (e.g., mini-protein) or other types of peptides (e.g., circular peptides) that would have a better chance to block RBD-ACE2 binding (Pomplun, 2020).

## Conclusion

For the great endanger of the COVID-19 pandemic to the human, any strategies or approaches that might lead to the discovery of therapeutics or cures deserve a try (Singh et al., 2021; Vivekanandhan et al., 2021). We utilized PhD to generate two 12-aa peptides with the potentials to inhibit S-protein bindings onto cellular ACE2. Structural modeling revealed that one (C6) might take effect by binding onto the S-RBD-ACE2 interaction face. More efforts should be made to improve the binding affinity of the peptides for S proteins, such as by modifying or transforming them into other types of molecules to block S protein-ACE2 adherence more efficiently. Ultimately, such peptides or their derivatives might be developed into therapeutics that block virus-host attachment and hinder disease onset.

## Data availability statement

The raw data supporting the conclusions of this article will be made available by the authors, without undue reservation.

## Author contributions

YQW conceived and designed the study. TW, JX, YLW, and WZ performed the experiments. YX and QY supplied reagents. BW performed the 3D structural modeling. TW, JX, and YQW analyzed the data and prepared the manuscript. All authors contributed to the article and approved the submitted version.
