# Supplementary material for: Receptor-binding domain-anchored peptides block binding of severe acute respiratory syndrome coronavirus 2 spike proteins with cell surface angiotensin-converting enzyme 2
Source: Front Microbiol. 2022 Sep 13;13:910343. doi: 10.3389/fmicb.2022.910343 (PMC9513850; doi:10.3389/fmicb.2022.910343)
Supplement: Supplementary file 1 [file Presentation_1.pdf]

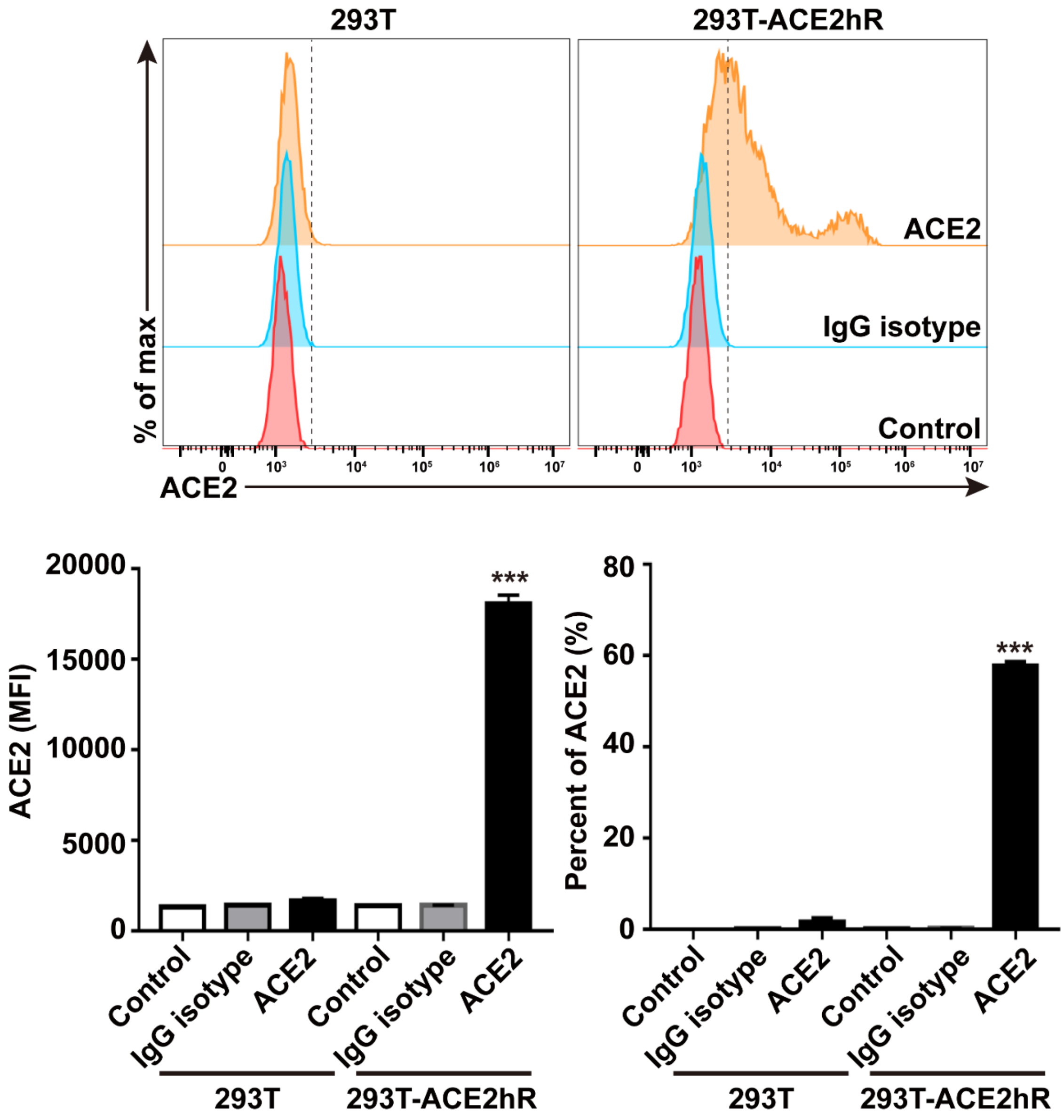

Figure S1. The measurement of hACE2 expression on 293T-ACE2hR cells or control 293T cells by flow cytometry. The single-cell suspensions were treated with rabbit anti-ACE2 (ab272500, at 1:250 dilution) for 30 min and labeled using fluorescence-conjugated anti-rabbit IgG (1:500) for another 30 min. Washes were applied between each step as routine. The cells were run on a flow cytometer, and the collected data were analyzed as stated in Materials and Methods. It could be told that hACE2 was abundantly expressed in most cultured 293T-ACE2hR cells.

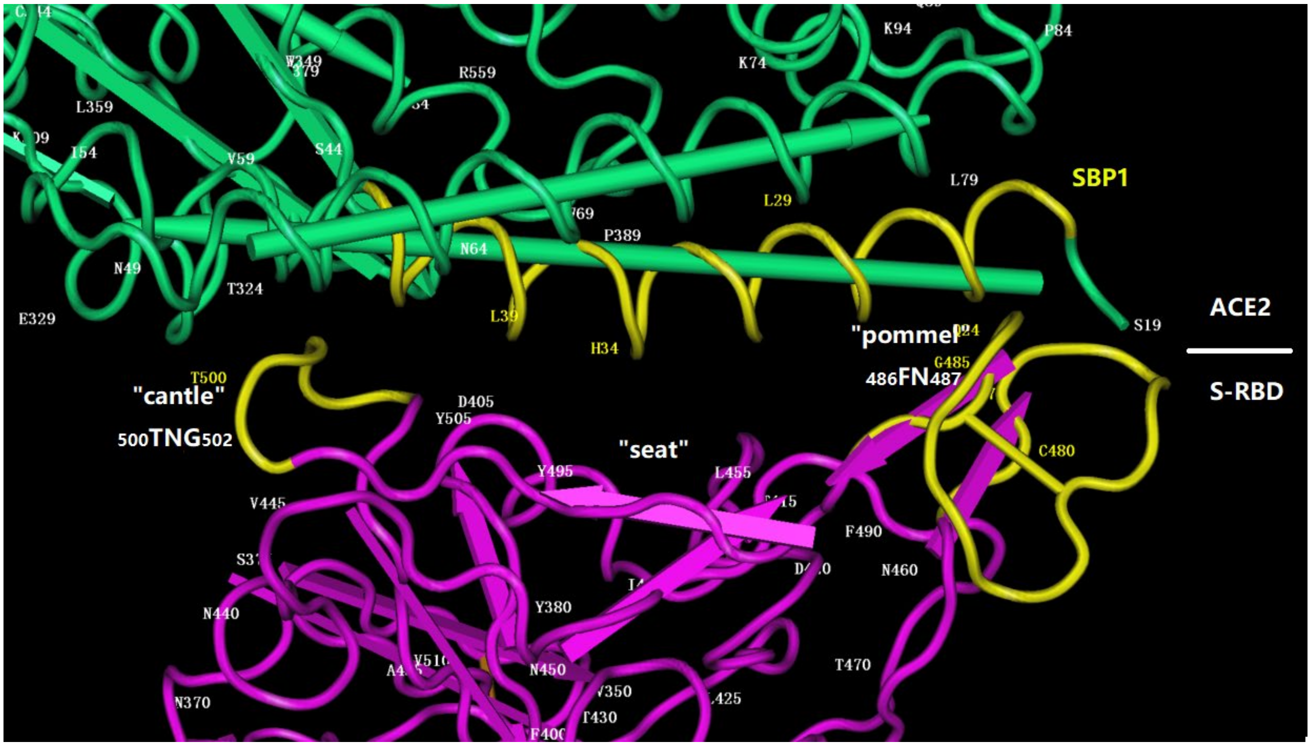

Figure S2. Part of the S-RBD-ACE2 interface shows the saddle shape of receptor binding motif and their counterpart residues on ACE2. The "cantle" and "pommel" sections were centered around $T_{500}N_{501}G_{502}$ and $F_{486}N_{487}$, respectively, and the sequence between them formed the "seat". SBP1 sequence of ACE2 was shown in goldenrod color, while SBP2 was the middle half of SBP1.
